# Adenomyoepithelial tumours and myoepithelial carcinomas of the breast – a spectrum of monophasic and biphasic tumours dominated by immature myoepithelial cells

**DOI:** 10.1186/1471-2407-5-92

**Published:** 2005-07-28

**Authors:** Daniela Hungermann, Horst Buerger, Christian Oehlschlegel, Hermann Herbst, Werner Boecker

**Affiliations:** 1 Institute of Pathology, Muenster University Hospital, Domagkstrasse 17, 48149 Muenster, Germany; 2 Institute of Pathology, Kantonsspital St. Gallen, Rorschacher Str. 95, CH-9007 St. Gallen, Switzerland; 3 Institute of Pathology, Region Süd, Klinikum Neukölln, Rudower Str. 48, 12351 Berlin, Germany

## Abstract

**Background:**

Adenomyoepithelial tumours and myoepithelial carcinomas of the breast are primarily defined by the presence of neoplastic cells with a myoepithelial immunophenotype. Current classification schemes are based on purely descriptive features and an assessment of individual prognosis is still problematic.

**Methods:**

A series of 27 adenomyoepithelial tumours of the breast was analysed immunohistochemically with antibodies directed against various cytokeratins, p63, smooth muscle alpha-actin (SMA) and vimentin. Additionally, double immunofluorescence and comparative genomic hybridisation (CGH) was performed.

**Results:**

Immunohistochemically, all the tumours showed a constant expression of high molecular weight cytokeratins (Ck) Ck5 and Ck14, p63, SMA and vimentin. With exception of one case diagnosed as myoepithelial carcinoma, all tested tumours expressed low molecular weight cytokeratin Ck18 in variable proportions of cells. Even in monophasic tumours lacking obvious glandular differentiation in conventional staining, a number of neoplastic cells still expressed those cytokeratins. Double immunofluorescence revealed tumour cells exclusively staining for Ck5/Ck14 in the presence of other cell populations that co-expressed high molecular weight Ck5/Ck14 as well as either low molecular weight Ck8/18 or SMA. Based on morphology, we assigned the series to three categories, benign, borderline and malignant. This classification was supported by a stepwise increase in cytogenetic alterations on CGH.

**Conclusion:**

Adenomyoepithelial tumours comprise a spectrum of neoplasms consisting of an admixture of glandular and myoepithelial differentiation patterns. As a key component SMA-positive cells co-expressing cytokeratins could be identified. Although categorisation of adenomyoepithelial tumours in benign, borderline and malignant was supported by results of CGH, any assessment of prognosis requires to be firmly based on morphological grounds. At present it is not yet clear, if and to what extent proposed Ck5-positive progenitor cells contribute to the immunohistochemical and morphological heterogeneity of these neoplasms of the breast.

## Background

Adenomyoepithelial tumours are a widely accepted, yet poorly understood diagnostic entity of breast neoplasms, the definition of which is based on cytological and architectural features. According to the most recent WHO classification, the spectrum of tumours included under this heading ranges from organoid, bi-phasic tumors with a combination of easily recognisable tubular formations and multi-layered spindle-celled myoepithelial elements to monophasic lesions with "mesenchymal" appearance [1-3].

In tumours of monophasic "mesenchymal" appearance, a potentially glandular differentiation can sometimes only be identified by staining for low-molecular-weight cytokeratins (Ck) Ck8/18. Malignant tumours without obvious morphological and immunophenotypic glandular features are currently classified as malignant myoepithelioma or myoepithelial carcinoma. These malignancies are characterized by infiltrating proliferations of atypical spindle cells with high mitotic activity, often displaying a dot-like cytoplasmic expression of smooth muscle α-actin (SMA), vimentin and Ck14. Because of this phenotypic heterogeneity, a specific position within the spectrum of proliferative breast lesions has not been assigned to adenomyoepithelial neoplasms, and unequivocal criteria for prognostic assessment have not yet been established [4,5].

We studied a series of 27 adenomyoepithelial tumours by comparative genomic hybridisation (CGH), double immunofluorescence and immunostaining for Ck5, Ck14, Ck8/18 and Ck19, as well as for vimentin, p63, SMA and the proliferation antigen detected by the monoclonal antibody MIB-1. Our data indicates that adenomyoepithelial tumours represent a spectrum of lesions that constantly express Ck5 and alternately show glandular and myoepithelial elements.

## Methods

### Tissue blocks

Tissue blocks of 27 cases, most of them referral cases, were selected from the files of the Institutes of Pathology of Muenster University Hospital and Kantonsspital St. Gallen. The tissue material had been fixed in neutral buffered formalin and paraffin-embedded according to standard procedures. Diagnoses were based on morphologic criteria using haematoxylin and eosin (H&E)-stained sections and immunohistology for Ck5 and Ck14, Ck8/18 and sm-actin. The clinical data, as far as available, is summarised in Table 1. The median age of patients presenting with a benign adenomyoepithelial tumour was 51 years, all patients were female. The median age of the patients with malignant adenomyoepithelial tumours was 53 years. Tumours classified as benign comprised a broad spectrum of histological differentiation ranging from biphasic lesions with well definable tubular structures to monophasic types, whereas almost all malignant tumours displayed a "mesenchymal" appearance. In order to be classified as benign, adenomyoepithelial tumours had to fulfil the following criteria: benign cytomorphology, a mitotic rate of less than 15 %, and absence of invasive features. Malignancy was diagnosed when the following criteria were met: a mitotic rate exceeding 15 % and/or malignant cytomorphology and/or invasive growth pattern. Those cases that did not fit either group were regarded as borderline tumours.

**Table 1:** Clinical data and results of conventional histology and immunohistology of adenomyoepithelial tumours of the breast.

**Case**	**age**	**Diagnosis**	**Pleomorphism**	**Necrosis**	**Tumour border**	**CK14**	**Ck19**	**Ck18**	**CD117**	**Vim**	**P63**	**Ck5/6**	**S100**	**KL-1**	**SMA**	**MIB-1[%]**	**ER**	**PR**	**Mitoses/ 10HPF**
1	42	benign, biphasic AM/ tubular	0	0	s	(+++)	(++)	(++)	(++)	(+)	(+++)	(+++)	nd	(+)	(+++)	2	(+)	(+)	0
2	57	benign, biphasic AM/ tubular/ spindle-cellular	(+)	0	s	(+++)	(+++)	(+++)	0	(+)	nd	(+++)	nd	(+++)	(+++)	5	(+++)	(++)	0
3	50	benign, biphasic AM/ tubular/ lobular	(+)	(+)	s	(++)	(0)	(+)	(++)	(++)	(+++)	(+++)	nd	(+)	(+)	10	0	0	1
4	75	benign, biphasic AM/ lobular	(+)	0	s	(+)	(++)	(++)	(+)	(+)	(+++)	(++)	nd	(+)	(++)	10	(+)	0	0
5	80	Benign, biphasic AM/ lobular	(+)	(+)	i	(++)	(+)	(+)	(++)	nd	(+++)	(++)	(+)	(+)	(++)	10	0	0	0
6	59	benign, biphasic AM/ tubular/ spindle-cellular	(+)	0	s	nd	nd	nd	nd	(+)	nd	(+++)	nd	(++)	(+++)	15	(+++)	0	1
7	65	benign, monophasic AM/ spindle-cellular	(+)	0	s	(+++)	0	(+++)	0	(+++)	nd	(+++)	(+++)	(+++)	(+++)	1	0	0	0
8	80	benign, biphasic AM/ tubular/ spindle-cellular	(+)	0	s	(+++)	(+++)	(+++)	(+)	(+)	nd	(++)	(++)	(++)	(++)	5	0	0	2
9	64	borderline, monophasic AM/ spindle-cellular	(+)	0	i	(+++)	0	(+)	(+)	(+++)	(+++)	(+++)	nd	(+)	(+++)	20	0	0	1
10	78	borderline, monophasic AM/ spindle-cellular	(++)	(+)	i	nd	nd	nd	nd	nd	(+)	(+++)	(+)	(+)	(+)	30	0	0	1
11	84	borderline, monophasic AM/ spindle-cellular	(+++)	0	i	(+++)	0	(+)	(+)	(+++)	nd	(+++)	nd	(+)	(++)	10	0	0	1
12	95	borderline, monophasic AM/ spindle-cellular	(++)	0	i	nd	nd	nd	nd	nd	nd	(+++)	(+)	(+)	(+)	20	0	0	1
13	66	borderline, monophasic AM/ spindle-cellular	(+++)	(+)	i	(+++)	0	(+)	(++)	(+++)	(+++)	(+)	nd	0	(+)	10	0	0	0
14	71	malignant, monophasic AM/ spindle-cellular	(+++)	(+)	i	(+++)	0	(+)	(+)	(+++)	(+++)	(++)	nd	(+)	(+)	20	0	0	1
15	68	malignant, monophasic AM/ spindle-cellular	(++)	(+)	i	(+++)	(++)	(+)	(+)	(+++)	(+++)	(+++)	(+)	(+)	(++)	40	0	0	12
16	93	malignant Myoepithelioma	(+++)	(+)	i	(+)	0	0	(++)	(+++)	(+++)	(+++)	nd	(+)	(++)	40	0	0	8
17	85	malignant, monophasic AM/ spindle-cellular	(+++)	(+)	i	(+++)	0	(+)	(+)	(+++)	(+++)	(+++)	nd	(+)	(++)	20	0	0	1
18	48	malignant, monophasic AM/ spindle-cellular	(++)	0	i	nd	nd	nd	nd	nd	nd	(+++)	nd	(+)	(+++)	25	0	0	6
19	?	malignant, monophasic AM/ spindle-cellular	(+++)	(+)	i	(+++)	(+)	(+)	0	(++)	(+++)	(+)	(+)	(+)	(+)	50	0	0	25
20	81	malignant, monophasic AM/ spindle-cellular	(+++)	(+)	i	nd	nd	nd	nd	nd	nd	(+++)	(+)	(+)	(+)	20	0	0	2
21	87	malignant, monophasic AM/ spindle-cellular	(+++)	(+)	i	(+++)	0	(+)	0	(+++)	nd	(+++)	0	(++)	(+++)	50	0	0	10
22	55	malignant, monophasic AM/ spindle-cellular	(+++)	(+)	i	(+++)	(+++)	(++)	(+)	(+++)	nd	(+++)	(+)	(+)	(+)	80	0	0	22
23	60	malignant, biphasic AM/ spindle-cellular	(+++)	(+++)	i	(+++)	(+)	(+++)	(++)	(+++)	nd	(+++)	(+++)	(+++)	(+++)	30	0	0	2
24	82	malignant, monophasic AM/ spindle-cellular	(+++)	(+++)	i	(+++)	(++)	(++)	(++)	0	nd	(+)	(+)	(+)	(+++)	75	0	0	12
25	57	malignant, monophasic AM/ spindle-cellular	(++)	(++)	i	(+++)	(+)	(+)	(+)	(+)	nd	(++)	(+)	(+)	(++)	70	0	0	62
26	45	malignant, monophasic AM/ spindle-cellular	(+++)	(+++)	i	(+)	(+++)	(++)	(++)	(++)	nd	(++)	(+++)	(+)	(++)	30	0	0	7
27	45	malignant, monophasic AM/ spindle-cellular	(++)	(+++)	i	(+)	(+++)	(+++)	(++)	(++)	nd	(+)	(+)	(+++)	(++)	60	0	0	11

S = sharply bordered lesions, i = infiltrative growth pattern

### Immunohistology

Paraffin sections of 8 benign, 5 borderline-type and 14 malignant adenomyoepithelial tumours were stained with antibodies (Table 2) using LSAB/ HRP (Ventana Nexes, Ventana Medical Systems, Tuscon, USA) or the LSAB/ AP (DAKO-Autostainer, DAKO Hamburg, Germany) techniques with DAB or Red as substrates, respectively.

**Table 2:** Antibodies and immunohistological staining procedures

Antigen	Antibody	Source	Dilution	Detection	Pretreatment
Vimentin	mAb V9	DAKO, Glostrup, Denmark	1:1000	LSAB/ HRP	Steamer
Ki-67 nuclear antigen	mAb MIB-1	DAKO, Glostrup, Denmark	1:100	LSAB/AP	Steamer
broad spectrum Cks	mAb KL-1	Immunotech, Marseille, France	1:50	LSAB/ HRP	Steamer
Ck5/6	mAb D5/16B4	Zymed, San Francisco, USA	1:50	LSAB/ AP	Steamer
Ck14	mAb Ll002	dianova GmbH, Hamburg, Germany	1:50	LSAB/ AP	Steamer
Ck8/18	mAb 5D3	dianova GmbH, Hamburg, Germany	1:40	LSAB/ AP	Steamer
Ck19	mAb RCK108	DAKO, Glostrup, Denmark	1:80	LSAB/ AP	Steamer
P63	mAb 4A4	DAKO, Glostrup, Denmark	1:100	LSAB/ AP	Steamer
Sm-α-actin (SMA)	mAb 1A4	DAKO, Glostrup, Denmark	1:3000	LSAB/ AP	Steamer
S-100 protein	Rabbit, polyclonal	DAKO, Glostrup, Denmark	1:1000	LSAB/ HRP	-
Estrogen receptor	mAb 6F11	Novocastra, Newcastle upon Tyne, UK	1:200	LSAB/ AP	Steamer
Progesterone receptor	mAb 16	Novocastra, Newcastle upon Tyne, UK	1:1000	LSAB/ AP	Steamer
c-Kit	Rabbit, polyclonal	DAKO, Glostrup, Denmark	1:100	LSAB/ AP	Steamer

mAb, mouse monoclonal antibody

### Double immunofluorescence experiments

After incubation with a first primary monoclonal antibody (mAb) sections were washed and incubated with FITC-conjugated rabbit anti-mouse antibodies (Dianova, Hamburg, Germany; diluted 1 : 50). They were then incubated with the second primary mAb that had been biotinylated previously (ARK™ biotinylating kit; Dako). The biotin label was subsequently visualised with Cy3-conjugated streptavidin (Dianova, 1 : 200). SMA was detected using a directly FITC-conjugated antibody. Counterstaining was performed with DAPI (Sigma, 5 μg/ml Tris buffer) for 15 s, and specimens were mounted with Vectashield (Vector Laboratories, Burlingame, USA). Controls were incubated without primary antibody or with mouse IgG. All cases were also stained using conventional enzyme immunolabeling [6].

### Microscopy and image processing

Immunostained slides were examined using a Zeiss Axioplan 2 bright field/fluorescence microscope equipped with appropriate filters. Separate images for DAPI, Cy3, and FITC staining were captured digitally from double-stained specimens into colour-separated components using an AxioCam digital camera (Carl Zeiss, Göttingen, Germany) and AxioVision 2.05 multi-channel image processing (Carl Zeiss Vision GmbH, Hallbergmoos, Germany). The red (for Cy3), blue (for DAPI), and green (for FITC) components were merged and composed for further analysis.

### Microdissection and DNA extraction

Manual microdissection was performed with a minimum of ten serial sections of 10 μm thickness cut from the paraffin blocks of all of the benign and malignant adenomyepithelial tumour specimens. DNA extraction was performed according to standard protocols of proteinase K digestion (1 mg/ml) followed by phenol-chloroform extraction.

### Comparative genomic hybridisation (CGH)

As previously described [7,8] fluorescence labeling of 900 ng DNA extracted from microdissected tissue was carried out by standard nick-translation reactions with biotin-16-deoxy-uridine-triphosphate (dUTP; Boehringer Mannheim, Mannheim, Germany). 300 ng of reference DNA from a healthy female donor was labeled with digoxigenin-11-dUTP (Boehringer Mannheim).

Regional shifts of the fluorescence ratio profile exceeding the 1.5 threshold were rated as amplifications. Since the target metaphase slides occasionally showed false-positive signals at 1p32-pter, 16p and the entire chromosome 19, those regions were excluded from our analysis.

## Results

### Conventional histology

**Figure 1:**
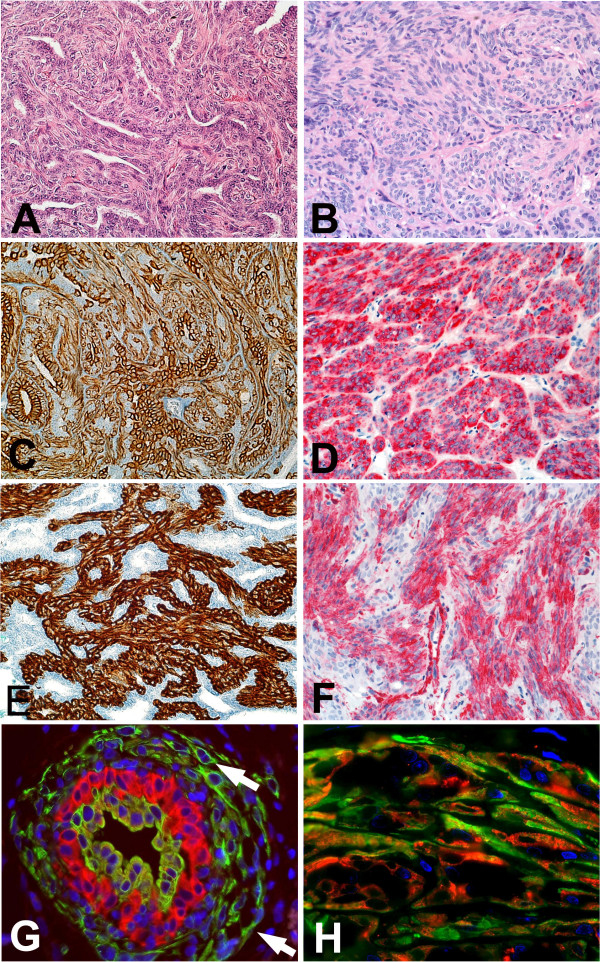
Conventional histology and immunostaining of two benign adenomyoepitheliomas of the breast (**A,C,E,G**: biphasic; **B,D,F**: monophasic). **A **shows a prominent tubular component, whereas in **B **a spindle-cell architecture predominates (A, H&E, magnification ×20, D, H&E, magnification ×20). **C **and **D **display distribution patterns of basal cytokeratin expression that are tantamount to the proportion of tumour cells (immunostaining for Ck5/6, magnification ×20) **E **and **F **show expression of sm α-actin (immunostaining for SMA, magnification ×20) with a distribution similar to normal breast tissue in biphasic benign adenomyoepithelioma. **G **shows double immunofluorescence staining (Ck5/6 green, Ck8/18 red, 40×) with prominent abluminal swarming of Ck5/6-positive cells. The proliferating glandular epithelium consists of differentiated (red signal) and less differentiated (hybrid signal) glandular cells. **H **shows double immunofluorescence staining (Ck5 red, SMA green, 63×) of a monophasic, malignant myoepithelial tumour with many cells coexpressing SMA and high molecular weigh Ck5. Note some less differentiated cells that express mainly Ck5 and some better differentiated cells with a predominance of SMA expression (green signal).

The adenomyoepithelial tumours analysed in this series displayed great morphological heterogeneity with variable growth patterns and cytological differentiations. All tumours showed at least foci of hyalinisation, sometimes in a basement membrane-like pattern with narrow eosin-red bands enmeshing the tumour cells, sometimes with prominent hyalinisation. Eight tumours were diagnosed as benign adenomyoepithelial tumours (Fig. 1, A, B) because they were architecturally inconspicuous and showed neither cytological atypia nor invasive growth. Five of these were biphasic with a combination of tubular structures and multilayered myoepithelial elements, whereas three showed additional areas of pure spindle-cell differentiation with bland cytomorphology. In only one of these a "lobular" growth pattern was found. Another two cases were characterized by a lobular growth pattern and one case by pure spindle cell differentiation. The growth fraction (MIB-1 labeling index) did not exceed 15 %.

**Figure 2:**
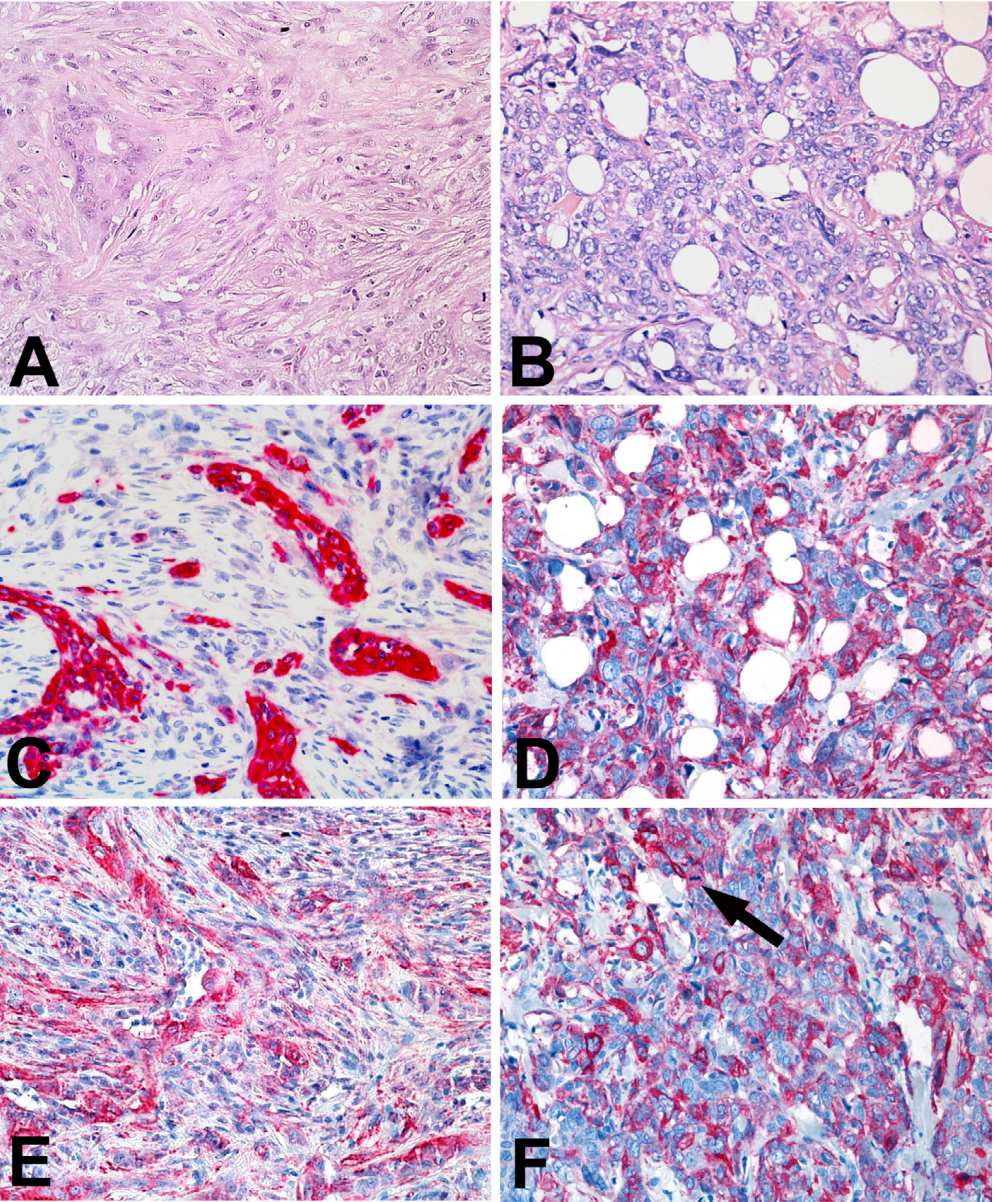
Conventional histology and immunostaining of two malignant adenomyoepitheliomas of the breast. **A, C and E **shows a biphasic malignant adenomyoepithelial tumour with distinct tubular formations, whereas **B, D and F **show a monophasic adenomyoepithelial carcinoma with diffuse infiltration of adipose tissue.(H&E, magnification ×20 **C **and **D **display distribution patterns of basal cytokeratin expression that are tantamount to the proportion of tumour cells (immunostaining for Ck5/6, magnification ×20). **E **and **F **show expression of SMA in a more irregular distribution compared to normal breast tissue in the malignant biphasic lesion and a rather diffuse pattern in monophasic lesions. A prominent mitotic figure is marked in F (arrow) (magnification ×20).

Fourteen adenomyoepithelial tumours were diagnosed as malignant (Fig. 2 A, B) in view of their cytological aspect, an infiltrative or destructive growth pattern and necrosis. The growth fraction exceeded 15 % in all those cases. These malignant adenomyoepithelial tumours often displayed a monophasic pattern consisting of spindle-shaped "mesenchymal" cells only. The cells were frequently arranged in a reticular, somewhat more cohesive pattern reminiscent of an abortive glandular differentiation. Other tumours showed an interwoven, storiform pattern and some displayed solid areas with an epitheloid or plasmacytoid aspect. Only one of these malignant tumours contained well-developed tubular structures and was diagnosed as biphasic malignant adenomyoepithelial tumour.

Five lesions could not be included in either of the above groups because they only showed some, but not all the malignant features. Diagnosed as borderline tumours, they displayed a conspicuous cytological variety with a growth fraction ranging from 10 to 30 % and, in some cases, focal necrosis. Irregularities in margin composition were not diagnosed as infiltrative growth. All the tumours of this group were predominantly of spindle-cell type. In six cases (22 %) of diverse prognostic groups metaplastic squamous cells were found to grow in small nests of keratinizing cells. Only one of the twenty-seven tumours which completely lacked glandular differentiation as well as Ck 8/18 expression was classified as pure myoepithelial carcinoma.

*Double immunofluorescence experiments *with two biphasic and three monophasic adeno-myoepithelial tumours yielded similar results. We found Ck5-positive tumour cells, glandular (Ck8/18+) and myoepithelial cells (SMA+), as well as cells co-expressing Ck5 and one of the lineage differentiation markers (either Ck5+, Ck8/18+ or Ck5+, SMA+) in all of these lesions (Fig 1, G, H).

### Immmunophenotype

All tumours tested for SMA expressed this antigen. With the exception of one case, all malignant adenomyoepithelial tumours showed reactivity with antibodies directed against vimentin. The high molecular weight cytokeratins Ck5 and Ck14 and SMA were variably expressed in all the tumours of this series. Furthermore, all the tumours tested were positive for p63 and, with the exception of one malignant neoplasm, all the lesions were at least focally positive for Ck8/18. Reactivity with the Ck19-specific antibody was noted in 5 of 7 (71 %) benign, in none of 3 borderline and in 8 of 12 malignant tumours. With the exception of a single case, all tumours showed at least focal reactivity with the antibody KL-1 directed against a broad spectrum of cytokeratins. 78 % of all the tumours tested displayed at least a weak reactivity with an antibody specific for CD117 (c-Kit).

### Comparative genomic hybridisation

**Figure 3:**
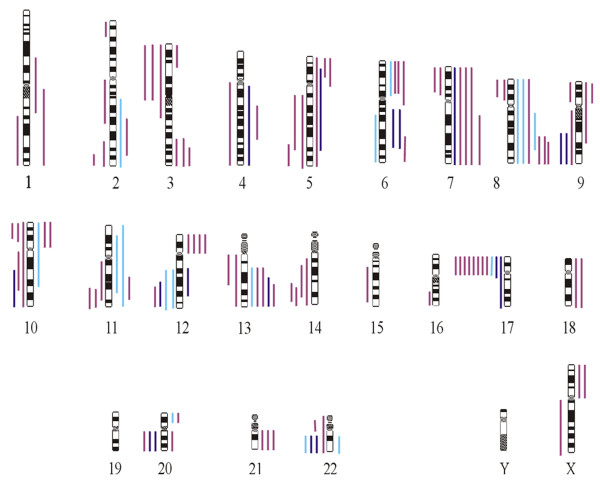
Summary of CGH profiles of 8 benign adenomyoepitheliomas (light blue), 5 adenomyoepitheliomas of borderline type (dark blue) and 14 adenomyoepithelial carcinomas (violet). A total of 131 gains and losses are distributed over 22 chromosomes with a preference of chromosomes 8, 17p and 13q.

In the group of benign adenomyoepithelial lesions, 5 of 7 tumours showed alterations comprising 13 gains and 5 losses of chromosomal regions (mean: 2.6). A total of 9 gains and 10 losses were found in 5 of the borderline tumours, while 3 tumours did not show any alterations (mean: 3.8). In 14 samples of malignant adenomyoepithelial tumours, a total of 94 DNA copy number changes were seen (mean: 6.7), while four malignant adenomyoepithelial tumours did not display any detectable changes (Fig. 3). With a total of 12, the changes on chromosome 8 were most frequent. Losses of 17p were detected in 6 malignant, 2 borderline and in 1 benign lesion. Less frequent alterations comprised gains of 13q (5 cases) and of the short arm of chromosomes 6 and 12, each observed in 4 cases.

Cases no. 8 and 24 were specimens obtained from a lesion diagnosed as benign adenomyoepithelial tumour, which nevertheless recurred two years later as a malignant tumour.

On CGH, the only alteration shared by both tumours was a gain of the long arm of chromosome 8, with amplification on this arm of chromosome 8 in the malignant recurrence.

We observed a stepwise, although statistically insignificant increase in the number of cytogenetic alterations per case. Their numbers were lowest for benign tumours, but steadily progressed in borderline and malignant adenomyoepithelial tumours. An elevated growth fraction correlated with an increase in the number of cytogenetic alterations (p = 0.07).

## Discussion

Tumours with an adenomyoepithelial differentiation span a broad and morphologically diverse spectrum of neoplasms ranging from patterns with biphasic glandular/myoepithelial architecture to monophasic pure spindle-cell variants. The biology of these rare tumours is poorly understood and their complex nature often gives rise to diagnostic uncertainty, especially when it comes to accurately assessing the prognosis of a given case. The hallmark of these tumours are SMA-positive neoplastic cells co-expressing cytokeratins in variable amounts. Furthermore, all of these tumours share a high proportion of cells that are reactive for basal-type Ck5/6 and Ck14 and p63 and most, even the monophasic ones, also contain tumour cells with expression of glandular type Ck8/18. This is in glaring contrast to myoepithelial carcinomas which are defined by a complete absence of glandular features. Nevertheless only one (case 16) of the spindle-cell tumours included in this series met the criteria of this category, all other tumours showed at least a small positive reaction with glandular-type cytokeratins. This would indicate that pure myoepithelial carcinomas are extremely rare. This admixture of glandular and myoepithelial components was also described in metaplastic spindle cell carcinoma, "fibromatosis-like" which has to be taken into account in differential diagnosis [9]. In view of our present morphological and immunohistochemical data, some of these tumours might be classified as adenomyoepithelial tumours.

Not unexpectedly, biphasic adenomyoepithelial lesions displayed a Ck8/18 expression that was confined to glandular structures and therefore similar to patterns present in normal breast tissue. Lesions with a monophasic "mesenchymal" appearance, however, displayed a Ck8/18 and Ck19-positive immunophenotype in spindle-cells often arranged in a reticular pattern (Figs 1 and 2. B, D, F). In addition, double immunofluorescence experiments showed co-expression of basal type keratins either in combination with glandular type keratins or SMA as a marker of the myoepithelial lineage. Part of the tumour cells solely expressed Ck5/6. Within the context of a recently published model of differentiation [6,10,11] in normal breast tissue this data would further point towards the existence of Ck5-positive progenitor cells with multi-lineage differentiation potential [12-16]. The potential importance of these presumed progenitor cells has already been shown in mouse tumours with both myoepithelial and glandular differentiation [17].

The assumption that adenomyoepithelial neoplasms are progenitor cell-derived lesions would help to explain the mixed glandular and myoepithelial architecture in the biphasic tumours as well as the bilinear immunophenotype in monophasic lesions. It seems that the tumour cells retain at least some functional remnants of their physiological counterparts. These include basement membrane formation of hemidesmosomes and laminin-binding sites and a strong polarity [18-21]. In the context of this hypothesis, the typical architectural element of adenomyoepithelial tumours, namely the predominant multilayered myoepithelial component with its swarming aspect and enclosed tubules or purely reticular pattern, can be attributed to an immature myoepithelial phenotype and function. Especially the latter may trigger a loss of polarity which leads to an inappropriate sorting of the myoepithelial cells.

From a clinical point of view, no clear criteria for the assessment of patient prognosis have been defined yet. Because these tumours are so rare, available data concerning the prognosis of these tumours is scarce. In accordance with reports in the literature [4,23], we applied criteria commonly used for assessing malignancy of these tumours which unfortunately were shown to be lacking in precision. Mitotic rates of up to 16 mitoses per 10 HPF were reported in lesions categorised as benign, whereas in contrast tumours with less than 3 mitoses per 10 HPF were diagnosed as malignant. Additionally, ill-defined margins and infiltrative growth patterns are observed in tumours of this entity, although it was never conclusively decided whether such features are indicative of a malignant outcome or a remnant of normal behaviour of myoepithelial cells as already documented *in vitro *[21].

Given this information, we propose a third category of tumours of uncertain prognosis in addition to a benign and a malignant category. The CGH analyses of our series of adenomyoepithelial neoplasms support this idea, because a stepwise increase in the average number of genetic alterations was observed. Growing numbers of genetic alterations are also associated with an increasing growth fraction (>10 %) However, this suggests a continuum in the progression of these tumours and is hardly helpful in the assessment of individual cases. For example, in our series 4 of 14 lesions with features of malignancy did not display any gross genetic alterations. This lack of practical applicability was painfully obvious in the case of an 80-year-old woman (no. 8 and 24) with an incompletely removed lesion described as benign adenomyoepithelial tumour. This apparently benign tumour recurred two years later as an overtly malignant adenomyoepithelioma. The initial tumour showed gains of genetic material in three distinct loci including 8q, whereas the malignant recurrence showed a further amplification on the long arm of chromosome 8 in addition to a loss of genetic material on the long arm of chromosomes 11 and 12. This suggests a clonal progression of changes already present in the initial lesion. Yet, similar to myoepithelial tumours of the salivary glands [24], a specific alteration pattern could not be defined for their counterparts of the breast, a result which is nevertheless in line with most published work in the field.

## Conclusion

Our data suggests the use of a two-tiered terminology according to which adenomyoepithelial tumours are classified as biphasic and monophasic in terms of their deviation from the normal architecture of the myoepithelial-epithelial formation. Furthermore, our data revealed hints for Ck5/6-positive cells as a key component in the histiogenesis of these neoplasms. Nevertheless, any prognostic assessment has to be made on morphological grounds with special attention to proliferative activity.

## Competing interests

The author(s) declare that they have no competing interests.

## Authors' contributions

DH analysed and interpreted the data and drafted the manuscript. HB made substantial contributions to the interpretation of the data. CO contributed tissue material and patient data as well as critically revising the diagnoses. HH scrutinised the manuscript, expressed constructive criticism and made most valuable suggestions. WB provided the theoretical framework, supervised the analysis of the data and corrected the manuscript.

## Pre-publication history

The pre-publication history for this paper can be accessed here:


